# Treatment with pollen allergen immunotherapy improves health-related quality of life in children and adolescents: a three-year follow-up-study

**DOI:** 10.1186/s13223-023-00756-9

**Published:** 2023-01-17

**Authors:** Helena Agenäs, Anna Lena Brorsson, Inger Kull, Anna Lindholm-Olinder

**Affiliations:** 1 Department of Clinical Science and Education, Södersjukhuset, Karolinska Institute, Sjukhusbacken 10, 118 83 Stockholm, Sweden; 2 Sachs’ Children and Youth Hospital, Södersjukhuset, Stockholm, Sweden; 3 Department of Neurobiology, Care Sciences and Society, Karolinska Institute, Stockholm, Sweden

**Keywords:** Allergic rhinoconjunctivitis, DISABKIDS, Health-related quality of life, Paediatric population, Subcutaneous immunotherapy

## Abstract

**Background:**

The immunological effect of allergen-specific immunotherapy is well documented, but few studies have examined the long-term effects of pollen subcutaneous immunotherapy (SCIT) on health-related quality of life (HRQoL) in children and adolescents. Therefore, the aims of this study were to evaluate the effect of pollen SCIT on HRQoL and to assess the association between HRQoL and symptoms among children and adolescents with allergic rhinoconjunctivitis in a 3-year follow-up.

**Methods:**

A prospective cohort study was conducted at a paediatric clinic in Sweden, including 158 children (5–16 years) on SCIT (birch and/or grass). Health-related quality of life, measured with DISABKIDS, symptom scores and allergen-specific IgE and IgG4 antibodies (blood test), were assessed at start, and after 1, 2 and 3 years of treatment. ANOVA and t-test were used to analyse differences over time, between groups and linear mixed model for the association between HRQoL and influencing factors.

**Results:**

After 1 year of pollen SCIT, HRQoL improved from 79.5 to 85.1 (p < 0.001), and the improvements were maintained (mean 1 years, 84.8, 3 years 87.2). Symptom scores decreased after 1 year, mean 19.9 to 11.5 (p < 0.001) and were maintained for year two (11.9) and year three (10.3). The proportion of children with severe or very severe symptoms decreased from 35.6% to 4.5% after 1 year of SCIT. Health-related quality of life was associated with symptoms at all measured timepoints (p = 0.001–0.031); higher symptom scores were associated with lower perceived HRQoL. Allergen-specific IgE antibodies decreased, birch from 151.0 to 76.8 kU/L (p < 0.001), and IgG4 antibodies increased, birch from 2.2 to 17.6 g/L (p < 0.001), grass from 0.5 to 14.3 g/L (p < 0.001), during the study period.

**Conclusion:**

After 1 year of pollen SCIT, HRQoL improved, and symptoms decreased; these changes were maintained during the study period. The proportion of severe and very severe symptoms significantly decreased.

## Key message

- Treatment with pollen allergen specific-immunotherapy improved health-related quality of life in children.
- Higher symptom scores were associated with lower perceived health-related quality of life.
- The proportion of children with severe or very severe symptoms from eyes, Nose and/or lungs had significantly decreased after 1 year of pollen SCIT and was maintained during the treatment years

## Introduction

Allergic rhinoconjunctivitis is a disease which usually begins as early as school age and has a major impact on quality of life and school attendance [1, 2]. Common symptoms of allergic rhinoconjunctivitis are itching, sneezing, runny nose, swollen and irritated eyes, itchy throat, and fatigue [1, 2]. The disease also impacts on the ability to concentrate, school performance, daily activities and sleep [3, 4]. Moreover, there is a link between allergic rhinoconjunctivitis and asthma [5]. Allergies and asthma have economic consequences for society, due to drug costs and absence from school or work [6].

In children and adolescents with allergic rhinoconjunctivitis, allergen-specific immunotherapy (AIT) is recommended when symptom control is not achieved with the help of conventional medication and environmental avoidance [7–9]. Allergen-specific immunotherapy with allergen extracts takes three to 4 years and is given in the form of subcutaneous injections (subcutaneous immunotherapy, SCIT) or with tablets under the tongue (sublingual immunotherapy, SLIT). In SCIT, the child receives injections up to three times a week during a build-up phase, followed by injections every 2 to 6 weeks over a period of years [7]. Allergen-specific immunotherapy prevents the occurrence of symptoms to the sensitizing allergen and reduces the need for medication [7, 10].

Symptoms of allergic rhinoconjunctivitis can significantly affect physical, psychological and social function [11]. Assessment of the severity of chronic diseases is based on symptom severity, but should also take account of the disease’s impact on quality of life [11]. The goal of treatment for children with allergies is that they should be able to live life as normally as possible and with high quality of life. Therefore, it is important to measure symptoms and health-related quality of life (HRQoL) directly from patients using patient-reported outcome measurements [12, 13]. Quality of life is defined by WHO as ‘[a] state of complete physical, mental and social well-being, and not merely the absence of disease or infirmity’ [14]. Health-related quality of life is usually used as a measure of self-perceived health, giving a clearer idea of how health affects quality of life [15].

Allergen-specific immunotherapy has been used for over a century and the immunological effect and decrease in symptoms and need of medication are well documented [7, 16]. However, few studies have evaluated the effect of pollen SCIT on HRQoL over time during childhood.

The aims of this study were to evaluate the effect of pollen SCIT on HRQoL, and to assess the association between HRQoL and symptoms among children and adolescents with allergic rhinoconjunctivitis in a 3-year follow-up.

## Methods

**Fig. 1:**
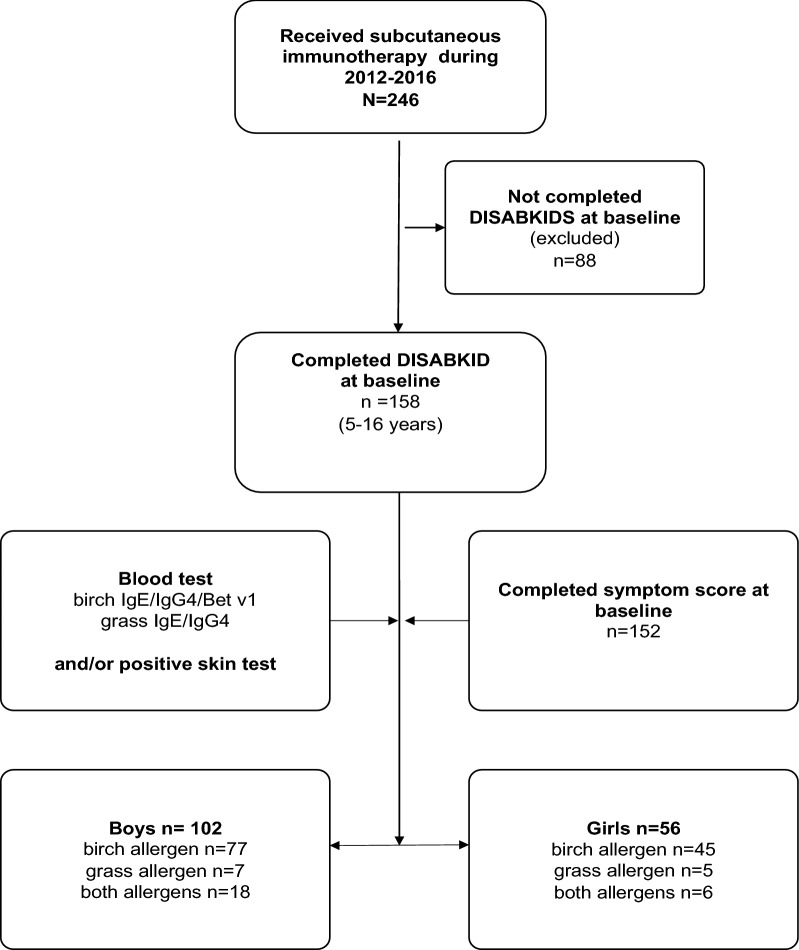
Flowchart recruitment process, showing total population and study population as well as number of boys and girls who participated in the study

This was a prospective cohort study conducted at a paediatric clinic in Stockholm, Sweden. Allergen-specific immunotherapy was offered to children and adolescents if their allergic rhinoconjunctivitis symptoms remained poorly controlled despite standard treatment. IgE sensitization was confirmed through blood test and/or skin tests. The immunotherapy for each allergen was given preseason (between September and January). If a local reaction occurred in connection with the injection, antihistamine was given in accordance with current guidelines [10]. During the years 2012–2016, 246 children and adolescents who received SCIT with birch and/or grass allergens were invited to the study (inclusion criteria). Of these, 158 children (5–16 years, mean age 11 years) completed the DISABKIDS questionnaires before start of pollen SCIT and were included in the current study (Fig. 1). There were no other exclusion criteria.

Health-related quality of life, measured with DISABKIDS, and symptom scores, were assessed before treatment start and after 1, 2 and 3 years of treatment. The questionnaires were distributed to the children after the pollen season (after September for gras and July for birch), in connection with visits to the hospital, and were answered by the children with help from their parents when required.

### Measurements

**Health-related quality of life** was measured using the validated generic instrument DISABKIDS for chronic conditions in children and adolescents [19, 20]. The generic form comprises 37 items assigned to six domains of HRQoL: independence, physical ability, inner strength, social inclusion, equality and treatment. The items were answered on a five-point Likert scale ranging from 1 (never) to 5 (always). Domain scores were transformed to a 0–100 scale, based on an equation in the creators’ instruction [19], with higher scores indicating higher quality of life. Total HRQoL was calculated as the mean of the six domains.

**Symptom scores** were used to measure symptoms from eyes, nose and/or lungs. Nasal symptoms included runny nose, stuffy nose, itchy nose, and sneezing. Eye symptoms included gravelly/red and watery eyes and lung symptoms included cough, wheezing, pressure over the chest and exercise-induced breathing difficulty. The severity of symptoms was assessed and scored on a four-point Likert scale (0 = none, 1 = moderate, 2 = severe, 3 = very severe). The sum of the symptom scores from nose (0–12), eyes (0–12) and lungs (0–12) was calculated, higher score indicates more symptom. If the total symptom score was above 24, the symptoms were considered severe or very severe.

Blood tests were drawn for the analysis of IgE sensitization, which involved measuring specific IgE and IgG4 antibodies against birch and grass pollen (ImmunoCAP System, Thermo Fisher/Phadia AB, Uppsala, Sweden). All participant except one, completed blood tests, one participant only underwent a skin prick test. The blood test and/or skin test was performed at start and after 1, 2 and 3 years of pollen SCIT treatment.

### Statistical analyses

Data were analysed using the statistical package SPSS (version 28). Demographic and categorical data are presented as numbers and percentages. Independent t-tests were used to determine significant differences between boys and girls and between age groups (0–10 and 11–18 years of age). Repeated measures ANOVA was used to analyse differences over time and the Bonferroni post hoc test was used to detect the location of differences. A linear mixed model was used to study associations between different domains of HRQoL, symptoms, time (before start of pollen SCIT and after 1, 2 and 3 years), sex and age. Toeplitz heterogeneous was used as the covariance structure, as it gave the best information criteria (-2 restricted log likelihood) [21]. The results were considered statistically significant if p < 0.05.

## Results

### Allergen-specific immunotherapy

The study population consisted of 158 children receiving pollen SCIT (boys n = 102 and girls n = 56). Ages at start were 5–16 years (mean 11, SD 2.7 years); mean age among girls was slightly higher than among boys (mean age 12.1, SD 2.5 vs. 11.0, SD 2.7 years of age, p = 0.012). All children were diagnosed with rhinoconjunctivitis and 74% (n = 117) had asthma. In total, 77% (n = 122) received SCIT with birch pollen allergen, 8% (n = 12) with grass pollen allergen and 15% (n = 24) with both allergens (Table 1).

**Table 1:** Demographics and clinical characteristics of total cohort and study population.

	Total population n = 246	Study population n = 158
Sex: boy n (%)	154 (63)	102 (65)
Age at start mean, SD (range)	11, 2.8 (5–17)	11, 2.7 (5–16)
Allergen treatment n (%)		
Birch	174 (71)	122 (77)
Grass	47 (19)	12 (8)
Both grass and birch	24 (10)	24 (15)
SYMPTOMS at start n (%)		
Rhinoconjunctivitis	68 (28)	41 (26)
Asthma and rhinoconjunctivitis	176 (71)	117 (74)

### HRQoL

After 1 year of pollen SCIT, there were statistically significant improvements in four of the six domains (independence from 80.3 to 86.3, p ≤ 0.001, inner strength from 81.7 to 89.0, p ≤ 0.001, equality from 87.0 to 91.7, p = 0.001 and physical ability from 67.0 to 74.7, p ≤ 0.001). The improvements were maintained during the study period (Table 2). The domain social inclusion showed improvement after 3 years of treatment (social inclusion baseline 81.6 vs. 87.8, p = 0.003). For the domain treatment there were no significant changes. Boys perceived their physical ability to be better than girls after 2 years of treatment (76.0 vs 67.5, p = 0.017) and after 3 years of treatment (82.8 vs 76.6, p = 0.036) (Table 2). Younger children (5–10 years) perceived their physical ability to be better than older children (79.4 vs 71.3, p = 0.01) after 1 year of treatment. There were no other statistically significant differences between the age groups.

**Table 2:** Description of HRQoL, measured with DISABKIDS, scores 0–100, before and after 1-3 years of SCIT

	Base mean (95% CI) n = 158 Girls, n = 56 Boys, n = 102	One year mean (95% CI) n = 124 Girls, n = 45 Boys, n = 79	Two years mean (95% CI) n = 125 Girls, n = 47 Boys, n = 78	Three years mean (95% CI) n = 111 Girls, n = 35 Boys, n = 76	p-value ^†^
Independence					
AIT group	80.3 (77.8–82.9)	86.3 (84.3–88.4)	86.1 (83.9–88.3)	87.8 (85.5–90.1)	< 0.001
Girl	81.7 (78.0–85.4)	85.0 (81.0–88.6)	84.4 (80.6–88.2)	90.2 (86.7–93.8)	0.001
Boy	79.6 (76.1–83.0)	87.3 (84.9–89.6)	87.1 (84.4–89.8)	86.7 (83.8–89.6)	< 0.001
Inner strength					
AIT group	81.7 (78.9–84.6)	89.0 (86.6–91.4)	87.0 (84.1–89.6)	90.6 (88.0–93.1)	< 0.001
Girl	82.5 (77.8–87.1)	86.7 (82.6–90.8)	85.7 (81.2–90.2)	91.0 (86.9–94.9)	0.006
Boy	81.3 (77.7–85.0)	90.3 (87.3–93.4)	87.6 (84.0–91.2)	90.4 (87.0–93.7)	< 0.001
Social inclusion					
AIT group	81.6 (79.0–84.1)	85.6 (83.1–88.0)	86.8 (84.4–89.2)	87.8 (85.4–90.1)	< 0.001
Girl	83.3 (78.6–88.0)	88.2 (85.0–91.5)	87.8 (83.6–91.9)	89.4 (86.0–93.0)	0.073
Boy	80.6 (77.6–83.5)	84.0 (80.6–87.4)	86.2 (83.2–89.1)	87.0 (83.9–90.1)	0.018
Equality					
AIT group	87.0 (84.6–89.4)	91.7 (89.9–93.5)	91.3 (89.2–93.4)	92.6 (90.5–94.7)	< 0.001
Girl	88.0 (83.7–92.2)	90.1 (87.0–93.2)	89.1 (85.5–92.7)	94.4 (91.3–97.5)	0.057
Boy	86.5 (83.5–89.5)	92.6 (90.4–94.8)	92.6 (90.1–95.2)	91.7 (89.0–94.5)	< 0.001
Physical ability					
AIT group	67.0 (63.7–70.1)	74.7 (71.3–77.4)	72.8 (69.4–76.3)	80.8 (78.1–83.6)	< 0.001
Girl	65.2 (59.9–70.4)	71.8 (66.9–76.7)	67.5 (62.7–72.3)	76.6 (71.5–81.7)	0.001
Boy	67.9 (63.8–72.0)	75.9 (72.0–79.8)	76.0 (71.4–80.6)	82.8 (79.6–86.0)	< 0.001
Treatment					
AIT group	78.6 (75.0–85.1)	82.6 (79.3–85.9)	84.7 (81.9–87.6)	86.5 (83.6–89.4)	0.144
Girl	79.8 (74.7–84.8)	78.6 (73.0–84.2)	81.2 (76.2–86.3)	86.9 (82.1–91.6)	0.087
Boy	77.9 (73.2–82.7)	84.8 (80.7–88.9)	86.7 (83.3–90.2)	86.3 (82.6–90.0)	0.229
Total HRQoL					
AIT group	79.5 (77.3–81.7)	85.1 (83.3–87.0)	84.8 (82.7–86.8)	87.2 (84.9–89.6)	< 0.001
Girl	80.1 (76.8–83.4)	83.6 (80.4–86.8)	82.7 (79.4–86.0)	87.7 (84.0–91.4)	0.001
Boy	79.2 (76.2–82.2)	86.0 (83.7–88.3)	86.0 (83.4–88.6)	87.1 (84.1–90.0)	< 0.001

^†^Repeated measures ANOVA

### Symptom score

**Fig. 2:**
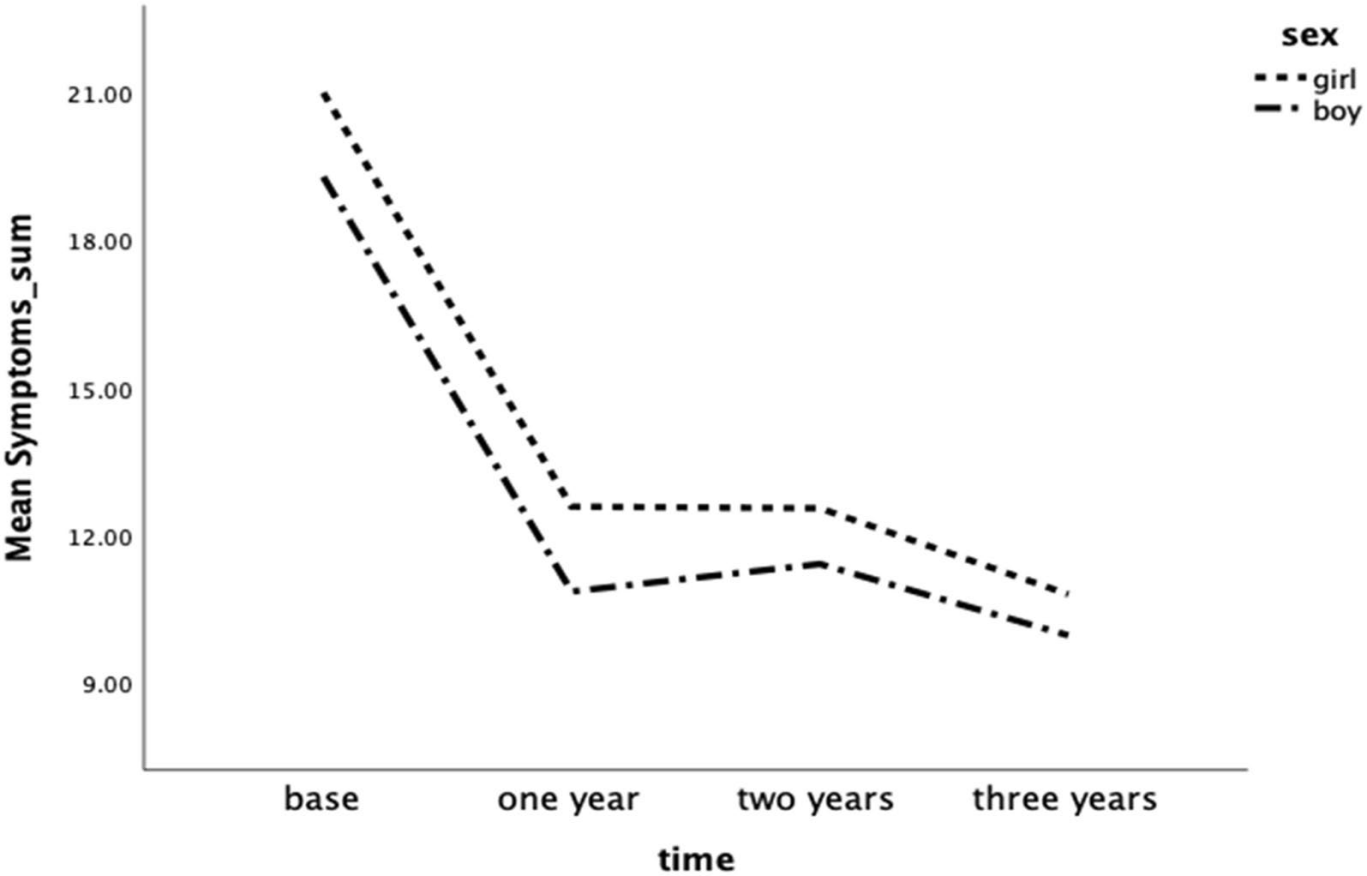
Descriptions of the effect on the mean of symptoms sum, from eyes, nose, and lungs, before start 1, 2 and 3 years after treatment with SCIT

Before start, 35.6% of the children had severe or very severe symptoms from eyes, nose, and/or lungs. After 1 year of pollen SCIT, the proportion decreased to 4.5%, after 2 years to 7% and after 3 years to 3.6%. In both girls and boys, the symptoms improved after 1 year of pollen SCIT, in eyes from mean 7.7 to 4.2 (p < 0.001), in nose from mean 7.8 to 5.0 (p < 0.001), and in lungs from mean 4.3 to 2.3 (p < 0.001). The improvements were maintained over the subsequent 2 years (eyes 2 years mean 4.4 and 3 years 4.0, nose 2 years mean 5.0 and 3 years 4.5, lungs 2 years mean 2.4 and 3 years 1.7). No significant differences in symptoms were observed between boys and girls during the treatment period. Older children (aged 11–15 years at start) reported more nasal symptoms after 1 year of pollen SCIT compared with younger children (5.6 vs. 4.2, p = 0.007). Older children also had a higher sum of symptoms than younger children (12.6 vs 9.8, p = 0.009) after 1 year. There were no other statistically significant differences between the age groups (Table 3, Fig. 2).

**Table 3:** Description of symptom scores from; eyes, nose, and lung^‡^, before and after allergen immunotherapy

Symptom from	Base mean (95% CI) n = 152 Girls, n = 53 Boys, n = 99 Age 5–10, n = 57 Age 11–16, n = 95	One year mean (95% CI) n = 134 Girls, n = 50 Boys, n = 84 Age 5–10, n = 51 Age 11–16, n = 83	Two years mean (95% CI) n = 128 Girls, n = 51 Boys, n = 77 Age 5–10, n = 42 Age 11–16, n = 86	Three years mean (95% CI) n = 85 Girls, n = 29 Boys, n = 56 Age 5–10, n = 33 Age 11–16, n = 52	p value^†^
Eyes					
All	7.7 (7.2–8.2)	4.2 (3.7–4.6)	4.4 (3.9–5.0)	4.0 (3.4-4.6)	< 0.001
Girls	8.4 (7.6–9.1)	4.5 (3.7–5.3)	4.6 (3.8–5.4)	4.6 (3.5–5.7)	< 0.001
Boys	7.3 (6.7–8.0)	4.0 (3.4–4.6)	4.3 (3.6–5.0)	3.7 (3.0–4.4)	< 0.001
Age 5–10 year	7.3 (6.4–8.1)	3.8 (3.2–4.4)	4.7 (3.8–5.6)	3.8 (2.9–4.7)	< 0.001
Age 11–16 year	8.0 (7.4–8.6)	4.4 (3.8–5.1)	4.3 (3.6–4.9)	4.2 (3.4–4.9)	< 0.001
Nose					
All	7.8 (7.3–8.3)	5.0 (4.6–5.6)	5.0 (4.6–5.6)	4.5 (4.0–5.1)	< 0.001
Girls	8.0 (7.3–8.7)	5.5 (4.6–6.3)	5.4 (4.6–6.2)	4.6 (3.6–5.5)	< 0.001
Boys	7.7 (7.1–8.4)	4.8 (4.2–5.4)	4.9 (4.2–5.5)	4.5 (3.8–5.2)	< 0.001
Age 5–10 year	7.7 (6.9–8.5)	4.2 (3.5–4.9)	4.6 (3.8–5.4)	4.2 (3.3–5.0)	< 0.001
Age 11–16 year	7.9 (7.3–8.5)	5.6 (4.9–6.2)	5.3 (4.7–5.9)	4.8 (4.0–5.5)	< 0.001
Lung					
All	4.3 (3.8–4.7)	2.3 (1.9–2.7)	2.4 (2.0–2.8)	1.7 (1.3–2.1)	< 0.001
Girls	4.6 (3.9–5.3)	2.6 (2.0–3.3)	2.6 (1.9–3.3)	1.5 (0.9–2.2)	< 0.001
Boys	4.1 (3.5–4.7)	2.1 (1.6–2.6)	2.3 (1.8–2.8)	1.8 (1.3–2.4)	< 0.001
Age 5–10 year	4.2 (3.3–5.8)	1.8 (1.2–2.3)	2.2 (1.5–3.0)	1.4 (0.9–2.0)	< 0.001
Age 11–16 year	4.3 (3.7–4.9)	2.6 (2.2–3.1)	2.5 (2.0–3.0)	1.9 (1.3–2.4)	< 0.001
Symptom’s sum					
All	19.9 (19.0–21.0)	11.5 (10.4–12.6)	11.9 (10.7–13.0)	10.3 (8.9–11.6)	< 0.001
Girls	21.0 (19.6–22.4)	12.6 (10.7–14.5)	12.5 (10.7–14.4)	10.7 (8.3–13.1)	< 0.001
Boys	19.3 (17.9–20.6)	10.8 (9.5–12.2)	11.4 (9.8–13.0)	10.0 (8.3–11.7)	< 0.001
Age 5–10 year	19.3 (17.6–20.9)	9.8 (8.3–11.2)	11.4 (9.4–13.5)	9.4 (7.2–11.5)	< 0.001
Age 11–16 year	20.2 (19.0–21.5)	12.6 (11.0–14.1)	12.1 (10.6–13.5)	10.8 (9.0–12.7)	< 0.001

^†^Repeated measures ANOVA

^‡^Eyes scores 0–12, nose scores 0–12, lung scores 0–12, higher score indicates more symptom

### Factors that influence HRQoL

The linear mixed model analyses showed that the different domains of HRQoL were associated with symptoms at all measured timepoints (p = 0.001–0.031), with higher symptom scores associated with lower perceived HRQoL. The domain physical ability was also associated with time (before start of pollen SCIT and after 1, 2 and 3 years, p = 0.002): physical ability increased over time. HRQoL was associated with gender. Boys perceived their physical ability to be better than girls did (p = 0.032). The domain treatment was also associated with time (p = 0.043): the impact of treatment on HRQoL (i.e., does the medication bother you; do you hate taking your medicine?) decreased over time with pollen SCIT.

### Allergen-specific IgE and IgG4 antibodies

IgE to birch decreased significantly during treatment (birch start 151.0 kU/L to 3 years 76.8 kU/L, p < 0.001). For grass, a decrease in IgE was observed, but it was not statistically significant (start 37.8 kU/L to 3 years 16.8 kU/L, p = 0.07). Also, there was an increase in IgG4 (birch start 2.2 g/L to 3 years 17.6 g/L, p < 0.001, grass start 0.5 g/L to 3 years 14.3 g/L, p < 0.001) (Table 4).

**Table 4:** Description of allergen specific antibodies, before, 1, 2 and 3 years of allergen immunotherapy

Allergen antibodies (kU_A_/L, g/L)	Base mean (95% CI)	One year mean (95% CI)	Two years mean (95% CI)	Three years mean (95% CI)	p value ^†^
IgE birch,	151.0 (113.5–188.6) n = 134	99.4 (77.9–120.8) n = 117	88.1 (66.2–110.0) n = 104	76.8 (57.0–96.7) n = 109	< 0.001
IgG4 birch	2.2 (0.9–3.4) n = 93	8.8 (6.9–10.7) n = 85	15.0 (12.2–17.9) n = 75	17.6 (14.8–20.5) n = 70	< 0.001
IgE grass	37.8 (4.9–70.8) n = 22	22.0 (8.8–35.2) n = 18	20.8 (7.5–34.2) n = 18	16.8 (5.9–27.6) n = 18	0.070
IgG4 grass	0.5 (0.1–0.9) n = 21	12.9 (8.1–17.7) n = 17	15.3 (8.5–22.2) n = 17	14.3 (7.5–21.1) n = 17	< 0.001

^†^Repeated measures ANOVA

## Discussion

Data from this prospective cohort study showed that pollen SCIT improved HRQoL, and reduced symptoms after 1 year of treatment and the improvements were maintained during the study period. The proportion of children with severe and/or very severe symptoms decreased significantly after 1 year of pollen SCIT. Moreover, there was an association between symptoms and the different domains of HRQoL: more symptoms were associated with decreased HRQoL. As expected, we could also see a decrease in IgE levels and an increase in IgG4 levels after 3 years of pollen SCIT.

The present study is one of few studies that has assessed long-term effects on symptoms and HRQoL of pollen SCIT in children. However, a recently published study examined children (5–15 years, n = 163) and adults (n = 236) treated with SLIT for grass pollen allergy. The results showed a good effect on both HRQoL and symptoms during the 3-year study period [22], which is in line with our study. Another long-term study, which compared the effects on HRQoL between SCIT and SLIT for children allergic to pollen or house dust mites, showed similar results after 3 years of immunotherapy [17]. In a study observing the effects of 1 year of pollen SCIT in children from age 12 years and adults with allergic rhinitis, HRQoL improved significantly, and asthmatic symptoms were reduced [18].

Changes in HRQoL may depend on age and maturity. Our study shows extensive improvement in HRQoL after 1 year, but it can be discussed if the aging process could play a role in this. However, we measured the effect of pollen SCIT in three different ways (HRQoL, symptoms and immunological factors), all of which showed an effect, which may indicate that neither age nor maturity affected the outcome, despite a long treatment period.

Our results with reduced symptoms after pollen SCIT are in line with the findings of a review study [16] that summarized data from 160 pollen SCIT and SLIT studies in children and adults (most adult participants) with allergic rhinoconjunctivitis and examined the symptom effects of pollen SCIT. Allergen-specific immunotherapy was found to be effective in decreasing symptoms and reducing medication needs in patients with allergic rhinoconjunctivitis during the treatment period [16].

In the present study, more boys than girls received pollen SCIT, and the girls were older when they started with the treatment, something seen in previous studies. Further, it has been reported that girls with allergic rhinitis are underdiagnosed and undertreated [17, 23]. This indicates that there is a need to investigate why there are gender differences in diagnosis and treatment.

In our study, having more symptoms affected HRQoL negatively. It is difficult to make a direct comparison with published literature, as there are no previously published reports on factors influencing HRQoL in connection with SCIT. A Swedish study examined how different allergic conditions affected HRQoL in children, showing that asthma significantly affected HRQoL and that allergic comorbidities also impacted HRQoL [24].

Allergen-specific immunotherapy requires commitment from the child and their family. This can be compared with treatment of other chronic diseases, such as oral immunotherapy in children with peanut allergy. In our study, HRQoL improved after pollen SCIT. In a randomized placebo-controlled study [25] which assessed, among other things, how quality of life was affected after oral immunotherapy with peanuts, a significant improvement in HRQoL was found after treatment completion [25].

### Strengths

The strengths of the present study include that it was performed in a clinical setting, evaluating effects of pollen SCIT over time and with a relatively large number of participants. The children started the treatment in different years, which decreased the impact of different pollen counts in different years. All children and adolescents received the same treatment, which was given in accordance with guidelines. In the present study, we used DISABKIDS, a validated instrument for assessing HRQoL in children with chronic diseases. It is considered suitable for capturing the emotional impact of the allergic disease, which can be difficult to do with disease-specific questionnaires [26–28].

### Limitations

A limitation of our study was the lack of a placebo control group; use of such a group was impossible for ethical reasons. Measurements of HRQoL based on life situations are difficult to evaluate as both subjective and objective circumstances must be considered. Measuring the effect of pollen SCIT in three different ways (HRQoL, symptoms, allergen-specific IgE and IgG4 antibodies) decreased the risk that the results depended on other circumstances.

DISABKIDS consists of 37 items, which may have affected the tendency to complete the questionnaire [19]. The younger children answered the questions with help of their parents when required, which may have affected the answers. To register symptoms from eyes, nose and/or lungs, we used symptom scores, which are not a validated instrument. Also, a limitation could be that we have no data on medication use during the time of immunotherapy.

## Conclusion

In children and adolescents with allergic rhinoconjunctivitis, pollen SCIT improved HRQoL and decreased symptom scores after 1 year of treatment, and the improvements were maintained during the study years. The proportion of severe and very severe symptoms significantly decreased after 1 year of pollen SCIT. Further there was a decrease in IgE levels and an increase in IgG4 levels after 3 years’ SCIT.

## Data Availability

The datasets used and/or analysed during the current study are available from the corresponding author on reasonable request.

## Abbreviations

AIT: Allergen-specific immunotherapy
HRQoL: Health-related quality of life
SCIT: Subcutaneous injections subcutaneous immunotherapy
SLIT: Sublingual immunotherapy
